# ANAC032 regulates root growth through the MYB30 gene regulatory network

**DOI:** 10.1038/s41598-019-47822-0

**Published:** 2019-08-06

**Authors:** Hiromasa Maki, Satomi Sakaoka, Tomotaka Itaya, Takamasa Suzuki, Kaho Mabuchi, Takashi Amabe, Nobutaka Suzuki, Tetsuya Higashiyama, Yasuomi Tada, Tsuyoshi Nakagawa, Atsushi Morikami, Hironaka Tsukagoshi

**Affiliations:** 1 Graduate School of Bioagricultural Science, Nagoya University, Furo-cho, Chikusa-ku, Nagoya, Aichi 464-8601 Japan; 2 Faculty of Agriculture, Meijo University, 1-501 Shiogamaguchi, Tempaku-ku, Nagoya, Aichi 468-8502 Japan; 3 Division of Biological Science, Graduate School of Science, Nagoya University, Furo-cho, Chikusa-ku, Nagoya, Aichi 464-8602 Japan; 4 Department of Biological Chemistry, College of Bioscience and Biotechnology, Chubu University, 1200 Matsumoto-cho, Kasugai, Aichi 478-8501 Japan; 5 Institute of Transformative Bio-Molecules (WPI-ITbM), Nagoya University, Furo-cho, Chikusa-ku, Nagoya, Aichi 464-8601 Japan; 6 Center for Gene Research, Nagoya University, Furo-cho, Chikusa-ku, Nagoya, Aichi 464-8602 Japan; 7 Department of Molecular and Functional Genomics, Interdisciplinary Center for Science Research, Organization for Research, Shimane University, Matsue, 690-8504 Japan

**Keywords:** Root apical meristem, Plant molecular biology, Plant signalling

## Abstract

Reactive oxygen species (ROS) play important roles as root growth regulators. We previously reported a comprehensive transcriptomic atlas, which we named ROS-map, that revealed ROS-responsible genes in *Arabidopsis* root tips. By using ROS-map, we have characterised an early ROS response key transcription factor, MYB30, as a regulator of root cell elongation under ROS signals. However, there are other ROS-responsible transcription factors which have the potential to regulate root growth. In the present study, we characterised the function of another early ROS-responsible transcription factor, ANAC032, that was selected from ROS-map. Overexpression of ANAC032 fused with the transcriptional activation domain, VP16, inhibited root growth, especially decreasing cell elongation. By transcriptome analysis, we revealed that ANAC032 regulated many stress-responsible genes in the roots. Intriguingly, ANAC032 upregulated *MYB30* and its target genes. The upregulation of MYB30 target genes was completely abolished in the *ANAC032-VP16x2* OX and ANAC032 estradiol-inducible line in *myb30-2* mutants. Moreover, root growth inhibition was alleviated in ANAC032-OX in *myb30-2* mutants. Overall, we characterised an upstream transcription factor, ANAC032, of the MYB30 transcriptional cascade which is a key regulator for root cell elongation under ROS signalling.

## Introduction

Plant roots are important organs for supporting the plant body because they absorb nutrients and water from soil and communicate with the surrounding environment. Therefore, root growth is crucial for the entire plant life cycle. Normally, plant root growth depends on the balance between cellular proliferation and differentiation at the root tip^1^. Many small molecules, such as plant hormones, regulate this balance through a gene regulatory network^2^. Under stressed conditions, however, plants regulate hormone amounts and plant hormones accelerate or decelerate root growth to counter environment stress^3^. In fact, plant hormones, such as auxin and cytokinin, regulate expression levels of several key transcription factor genes, and consequently these transcription factors drive the expression of their downstream target genes^4^. In turn, plant hormones act as signalling molecules for regulating plant root growth through complicated transcriptional regulatory networks.

As for the signal molecule that possibly regulates root growth, reactive oxygen species (ROS) are also important molecules. ROS regulates almost all aspects of root development ranging from the root meristematic zone to the maturation zone^5^. Interestingly, ROS serves key functions as regulator for multiple plant life events from cell wall synthesis to plant immunity^6^. In addition, ROS and other signalling molecules have complex and important molecular links that regulate plant growth and stress responses^7^. Under abiotic and biotic stresses, plants increase their levels of ROS to counter the effects of the stress.

Since plant roots are always exposed to multiple stresses from the soil, ROS regulation of root development is one of the most important roles among the diverse functions of ROS. Exogenous hydrogen peroxide (H_2_O_2_) treatment has been found to reduce *Arabidopsis* root growth^8,9^. We previously demonstrated a transcriptome analysis for elucidating the genes involved in root growth regulation in the presence of H_2_O_2_, which we named “ROS-map”^10^. We found several transcription factor genes that were differentially expressed after H_2_O_2_ treatment. Among them, a transcription factor, *MYB30*, significantly responded to H_2_O_2_ treatment and regulated cell elongation^10^. MYB30 directly regulated three genes, which encoded lipid transfer proteins. Moreover, these genes were induced by H_2_O_2_ treatment via MYB30, and expressed the same cell types as *MYB30*. However, there remains many genes for which expression changed under H_2_O_2_ treatment even in *myb30* mutants. This indicated that there were other transcriptional regulatory pathways for root development under ROS signalling.

The expression of one other transcription factor, *ANAC032*, was clearly induced in the ROS-map dataset, which prompted us to investigate its role further. The NAC (NAM, no apical meristem; ATAF1/2, *Arabidopsis* transcription activation factor; and CUC2, CUP-shaped cotyledon2) transcription factor family is a large gene family^11–13^, and has a range of functions from plant growth regulation to plant stress response^14–16^. Among them, ANAC032 has been characterised as a transcriptional regulator for age-dependent and stress-induced leaf senescence^17^. ANAC032 also promotes H_2_O_2_ production under auxin and salinity stresses. At the same time, *ANAC032* upregulated the expression of chlorophyll-degrading genes and senescence-associated genes. This study indicated that ANAC032 is involved in feed-forward regulation of leaf senescence. Moreover, ANAC032 negatively regulates the gene expression of anthocyanin-biosynthesizing genes in *Arabidopsis* in response to abiotic stress such as high-intensity light, salinity, and oxidation^18^. In addition, ANAC032 regulated several important transcription factors that are involved in plant immune response in the leaf^19^. Since ANAC032 activates salicylic acid (SA) signalling and at the same time downregulates genes responsible for jasmonic acid (JA), it is a key mediator between SA- and JA-dependent defence signalling. According to these previous studies, ANAC032 plays important roles in plant stress responses.

In the present study, we investigated the function of ANAC032 in primary root development, especially in young root development. From an analysis of transcriptome data and the *ANAC032* estradiol-inducible gene expression line, we found that *ANAC032* is an upstream of the *MYB30* regulatory gene network. We also reveal part of the transcriptional network for regulating root development, especially cell elongation, under ROS signalling that is controlled by ANAC032 through the MYB30 gene regulatory network.

## Results

### *ANAC032* in the elongation zone of root tips and its expression were induced under hydrogen peroxide treatment

**Figure 1:**
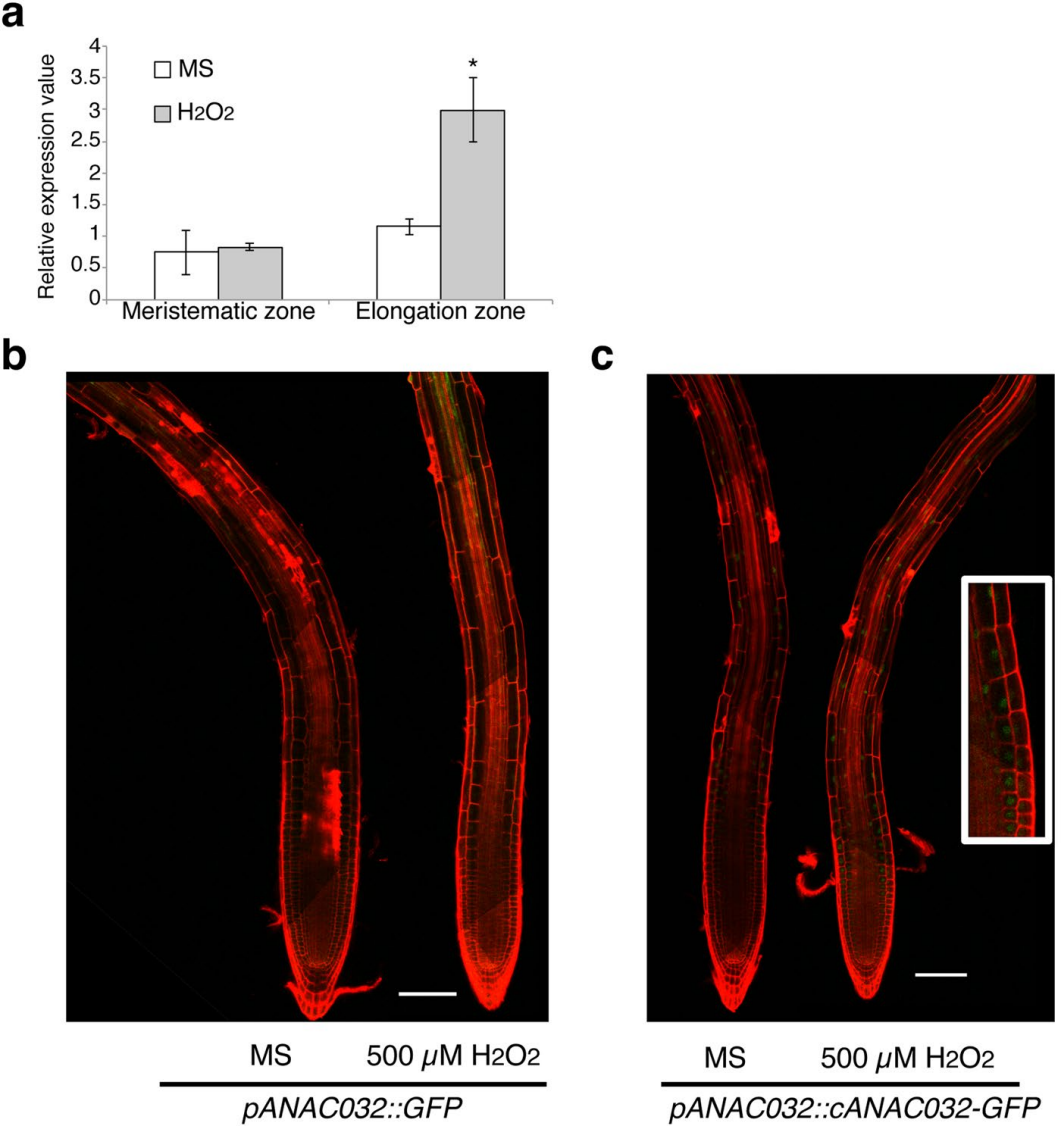
Expression pattern of ANAC032 in the root tip. (**a**) *ANAC032* expression in 5-day-old Col-0 root meristematic zone and the elongation zone treated with Murashige and Skoog (MS) agarose medium and 500 µM H_2_O_2_ containing agarose medium for 24 h (n = 3, means ± standard deviation [SD]). *p < 0.01, determined using Student’s *t*-test compared with the control. (**b**) Expression of 5-day-old *pANAC032::GFP* in Col-0 plants (transcriptional fusion) that were treated with MS agarose medium and 500 µM H_2_O_2_ containing agarose medium for 24 h. Roots were stained with propidium iodide (PI). Scale bar = 100 µm. (**c**) Expression of 5-day-old *pANAC032::ANAC032-GFP* in Col-0 plants (translational fusion) that were treated with MS agarose medium and 500 µM H_2_O_2_ containing agarose medium for 24 h. Roots were stained with PI. Scale bar = 100 µm. The inset image shows a higher magnification of the transition zone of *pANAC032::cANAC032-GFP* treated with 500 µM H_2_O_2_ agarose medium for 24 h.

We have previously reported that at least 50 transcription factors were upregulated by the hydrogen peroxide (H_2_O_2_) treatment at root tips within 6 h. Among them, we reported that MYB30 was one of the important root growth regulators under the H_2_O_2_ treatment^10^. Other than *MYB30*, we chose another transcription factor, *ANAC032*, that was also significantly induced within 1 h of H_2_O_2_ treatment. We initially investigated *ANAC032* expression level at the root tip using RT-qPCR. To investigate *ANAC032* response in proliferating and elongating cells, we cut *Arabidopsis* root tips into two zones, the meristematic and the elongation zones. *ANAC032* was significantly upregulated after 24 h of H_2_O_2_ treatment only in the elongation zone (Fig. 1a).

To obtain further details regarding *ANAC032* spatiotemporal expression patterns in the root, we observed green fluorescent protein (GFP) of *ANAC032* transcriptional (*pANAC032::GFP*) and translational (*pANAC032::ANAC032-GFP*) fusions. *pANAC032::GFP* showed GFP in the vascular tissues of the maturation zone and weak expression was detected in the elongation zone and epidermis (Fig. 1b). Though *pANAC032::GFP* expression was induced by the 500 µM H_2_O_2_ treatment in the vascular tissues and epidermis in the maturation zone, only weak induction of fluorescence was detected in the basal meristematic zone under H_2_O_2_ treatment (Fig. 1b and Supplementary Fig. S1). As for *pANAC032::ANAC032-GFP*, GFP was detected in the nucleolus of all cell types in the maturation zone and weak fluorescence was detected in the epidermis and cortex of the elongation zone similar to *pANAC032::GFP* (Fig. 1c). The expression of these fusions became stronger after H_2_O_2_ treatment (Supplementary Fig. S1). Moreover, GFP fluorescence could be detected in the epidermis, cortex, and endodermis from the basal meristematic zone to the elongation zone, or the transition zone (Supplementary Fig. S1). The GFP fluorescence in apical meristematic zone comes from lateral root cap after H_2_O_2_ treatment, not from the primary root cells. Although we used the same promoter region for both transcriptional and translational fusions, GFP expression of the translational fusions in the vascular tissue weakened. To assess in more detail the expression of *ANAC032* at the root tip, we took ten Z-stack images of the *pANAC032::ANAC032-GFP* root tips (Supplementary Fig. S1). From this, ANAC032-GFP was detected in the nucleus of epidermal cells in the transition zone, and its expression strengthened following H_2_O_2_ treatment. We also performed time-lapse imaging to observe ANAC032 expression along with root growth (Supplementary Movie S1 and S2). Similar to the results shown in Fig. 1c, ANAC032-GFP accumulated in the nucleus of the epidermal cell layers from the basal meristematic zone to the maturation zone under control conditions (Supplementary Movie S1). ANAC032 expression was promoted in the transition zone along with root growth after H_2_O_2_ treatment (Supplementary Movie S1). We also constructed a pseudo-colour integrative surface plot (Supplementary Movie S2), which showed strong intensity at the transition zone, especially 1 h and 11 h after initiating the imaging of ANAC032-GFP under the H_2_O_2_ treatment. These results suggest that ANAC032 plays a dominant role in the transition zone but not in the apical meristematic zone.

### ANAC032 decreased root growth

**Figure 2:**
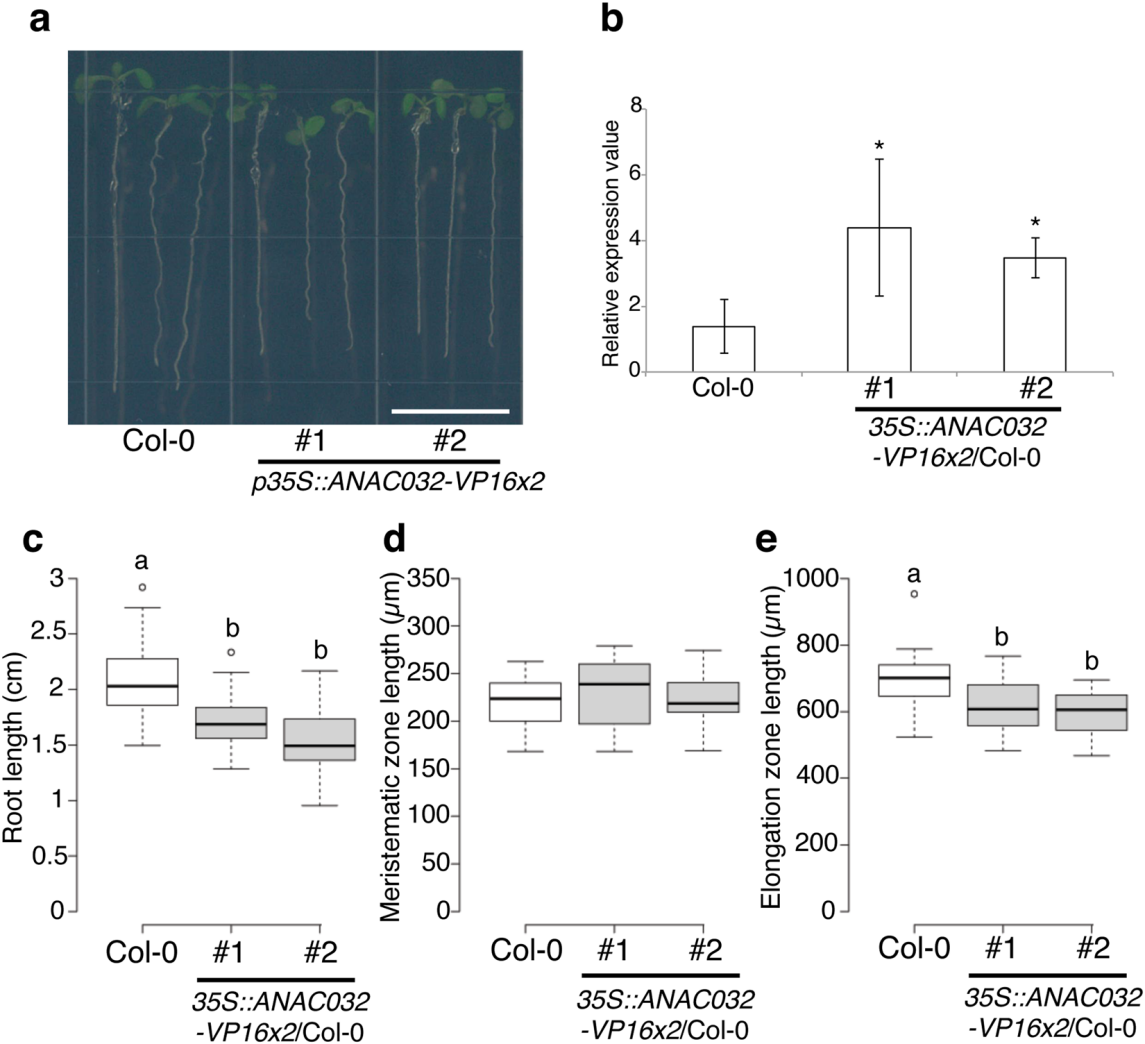
Root phenotype of *ANAC032-VP16x2* overexpression. (**a**) Representative images of 6-day-old plants of Col-0 and two independent lines of *35S::ANAC032-VP16x2*/Col-0. Scale bar = 1 cm. (**b**) RT-qPCR analysis of *ANAC032* expression in 6-day-old whole root of Col-0, and two independent lines of *35S::ANAC032-VP16x2*/Col-0 (#1 and #2) (n = 3, means ± SD); *p < 0.01, determined using Student’s *t*-test compared to Col-0. **(c**) Root length of 6-day-old plants of Col-0 (white box) and two independent *35S::ANAC032-VP16x2*/Col-0 lines (grey boxes) (n = 30, letters above boxes indicate statistically significant differences between samples as determined by Tukey’s HSD test (*p* < 0.05). (**d**) The meristematic zone length of 6-day-old plants of Col-0 (white box) and two independent *35S::ANAC032-VP16x2*/Col-0 lines (grey boxes) (n = 30). (**e**) The elongation zone length of 6-day-old plants of Col-0 (white box) and two independent *35S::ANAC032-VP16x2*/Col-0 lines (grey boxes) (n = 30, letters above boxes indicated statistically significant differences between samples as determined by Tukey’s HSD test (*p* < 0.05)).

To investigate if ANAC032 regulates root growth positively or negatively, we first obtained the T-DNA insertion line of *ANAC032* from the *Arabidopsis* Biological Resource Center (ABRC). We confirmed that the T-DNA insertion line SALK_012253 was homozygous by PCR-based genotyping. Although the full length of *ANAC032* mRNA was hardly detected by RT-PCR in this T-DNA insertion line, the root length of the T-DNA insertion mutant under both control and H_2_O_2_ treatment was comparable to that of the wild type (Supplementary Fig. S2). Since we hypothesised that the NAC domain containing proteins would have possible functional redundancy with each other owing to the large size of the gene family^20^, we decided to use the constitutive overexpression of *ANAC032* that fused with VP16x2, repeating the VP16 transcriptional activation domain twice, driving 35S promoter, instead of analysing the T-DNA insertion mutant. *ANAC032-VP16x2* OX plants had shorter roots than wild-type plant (Fig. 2a) and the expression levels of *ANAC032* in the *ANAC032-VP16x2* OX were about four times those in the wild type (Fig. 2b). The expression level of *ANAC032* in *ANAC032-VP16x2* OX was not strong, but *VP16x2* has enough effect on ANAC032 to allow it to function in root development. We also measured the root length of the wild type and two lines of *ANAC032-VP16x2* OX (Fig. 2c). *ANAC032-VP16x2* OXs had significantly shorter roots than the wild type. To investigate the effect of *ANA032-VP16x2* OX at the root tip, we measured the length of the meristematic zone and the elongation zone (Fig. 2d,e). The meristematic zone length was comparable between the wild type and *ANAC032-VP16x2* OXs. By contrast, the elongation zone length of *ANAC032-VP16x2* OXs was significantly shorter than that of the wild type. These results represent original *ANAC032* expression patterns (Fig. 1b,c) and indicated that ANAC032 had function for cell elongation. These results also indicated that ANAC032 is a negative regulator of root growth.

**Figure 3:**
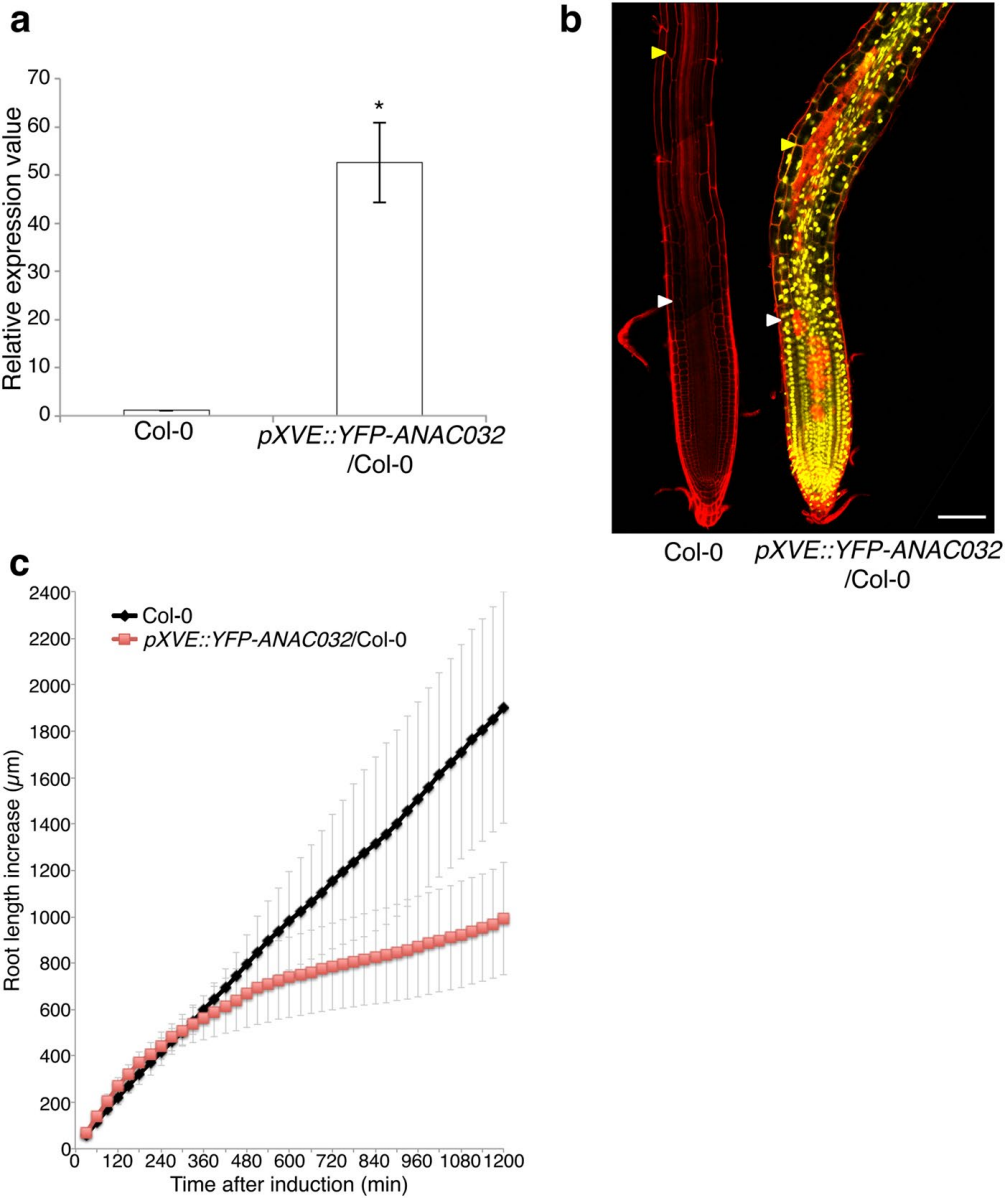
Root phenotype of the *ANAC032* estradiol-inducible line (*pXVE::YFP-ANAC032*). (**a**) RT-qPCR analysis of *ANAC032* in the 5-day-old whole root of Col-0 and *pXVE::YFP-ANAC032*/Col-0 that were treated with 5 µM estradiol containing agarose medium for 24 h. (n = 3, means ± SD); *p < 0.01, determined using Student’s *t*-test compared to Col-0. (**b**) Confocal microscope images of 5-day-old Col-0 and *pXVE::YFP-ANAC032*/Col-0 that were treated with 5 µM estradiol containing agarose medium for 24 h. Roots were stained with propidium iodide (PI). White arrowhead indicated the end of meristematic zone, and yellow arrowhead indicated the end of elongation zone. Scale bar = 100 µm. (**c**) Root length increase in 5-day-old roots of Col-0 (black diamonds) and *pXVE::YFP-ANAC032*/Col-0 (red squares) treated with 5 µM estradiol containing agarose medium. Root length increase was measured every 30 min after starting by time lapse imaging (n = 7, means ± SD).

To further test the more direct effects of ANAC032 overexpression on root growth, we constructed an estradiol-inducible gene expression version of *ANAC032* fused with yellow fluorescent protein (YFP) by cloning the *YFP-ANAC032* gene into pMDC7^21^. We first investigated the induction level of *ANAC032* after 24 h of 5 µM estradiol treatment (Fig. 3a) and found it to be over 50 times more than that in the wild type. We examined the roots of this induction line under a confocal microscope (Fig. 3b). After 24 h treatment with 5 µM estradiol, the induction lines had shorter elongation zones than those in the wild type. This result was consistent with the *ANAC032-VP16x2* OX root phenotype. Furthermore, we performed time-lapse imaging and measured root elongation every 30 min (Fig. 3c). Root elongation of the wild type and *pXVE::YFP-ANAC032* was almost the same until 360 min after 5 µM estradiol treatment. However, after 360 min root elongation was strongly inhibited in the *pXVE::YFP-ANAC032*. This result indicated that ANAC032 was a negative regulator of root growth.

### ANAC032 regulated a set of stress response genes

**Figure 4:**
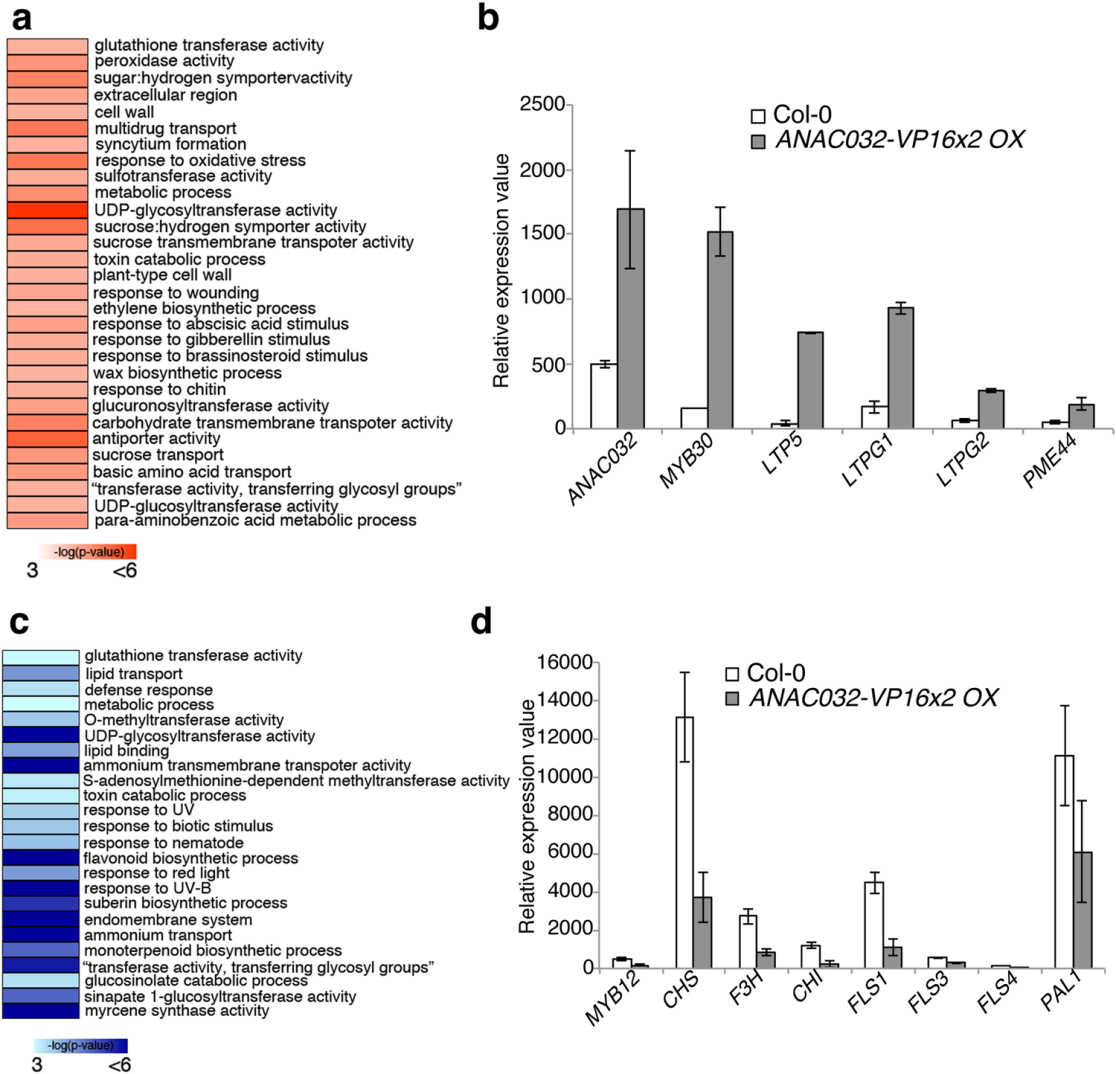
ANAC032-regulated genes in the root. (**a**) Enriched Gene Ontology (GO) categories within genes that were significantly upregulated in the *ANAC032-VP16x2* overexpression lines (*ANAC032-VP16x2* OX). (**b**) RNA expression of *ANAC032*, *MYB30*, and MYB30-target genes in Col-0 and *ANAC032-VP16x2* OX in the RNAseq analysis. (**c**) GO categories within genes that were significantly downregulated in *ANAC032-VP16x2* OX. (**d**) RNA expression of selected flavonoid biosynthesis related genes in Col-0 and *ANAC032-VP16x2* OX in the RNAseq analysis. Expression data were obtained from normalised RNAseq reads of Col-0 and *ANAC032-VP16x2* OX samples. White boxes: 6-day-old Col-0 whole root. Grey boxes: 6-day-old *ANAC032-VP16x2* OX whole root. n = 2, means ± SD.

To investigate ANAC032 regulation of gene expressions, we conducted an RNAseq analysis by using the *ANAC032-VP16x2* OX line. We found that 999 and 361 genes (singletons) were significantly upregulated and downregulated, respectively. We analysed the Gene Ontology (GO) categories of the upregulated genes in the *ANA032-VP16x2* OX line (Fig. 4a). We found that 30 GO categories were enriched. Among them, the most significantly enriched was “UDP-glycosyltransferase activity”. We also found categories related to plant hormone responses such as “abscisic acid (ABA) stimulus”, “gibberellin stimulus”, and “brassinosteroid stimulus”. Moreover, we identified categories related to extracellular components such as “cell wall”, “wax biosynthesis”, and “sugar metabolism”. Interestingly, among the upregulated genes in *ANAC032-VP16x2 OX*, we found *MYB30* and its target genes: *LTPG1*, *LTPG2*, *LTP5*, and *PME44* (Fig. 4b). We also compared the genes that were upregulated in both *ANAC032-VP16x2* OX and *MYB30* OX (Supplementary Fig. S3). 53 out of 203 genes that were upregulated in *MYB30* OX^10^ were also upregulated in *ANAC032-VP16x2* OX (Supplementary Table S1). The GO terms “lipid transport” and “wax biosynthesis” were enriched for 53 genes (Supplementary Fig. S3). This indicated that part of the MYB30 regulatory gene network could be under ANAC032 transcriptional regulation.

In contrast, there were 24 GO categories enriched among the downregulated genes (Fig. 4c). Among them, the most significantly enriched GO term was “flavonoid biosynthetic process”. We also found “UDP-glycosyltransferase activity” and “glutathione transferase activity” in the downregulated genes that we also found in the upregulated genes. GO categories related to stress response such as “UV”, “nematode”, and “defence” were also enriched. Focusing on the “flavonoid biosynthetic process” GO term, it has been previously reported that overexpression of *ANAC032* reduced anthocyanin levels in the shoots^18^. This was consistent with the results of our RNAseq analysis. We also determined the expressions of several genes involved in flavonoid biosynthesis, such as *MYB12*, *Chalcone Synthase* (*CHS*), *flavonol Synthase*, *flavanone 3-hydroxylase*, and *Chalcone flavanone isomerase* (*CHI*) from our RNAseq results (Fig. 4d). All of these genes were strongly downregulated in *ANAC032-VP16x2* OX. We also compared the genes that were downregulated in both *ANAC032-VP16x2* OX and *MYB30* OX (Supplementary Fig. S3). 43 out of 548 genes that were downregulated in *MYB30* OX^10^ were also downregulated in *ANAC032-VP16x2* OX (Supplementary Table S1.). However, there were no significantly enriched GO terms among the co-downregulated genes.

### The MYB30 regulatory network plays a role in root development under the ANAC032 regulatory network

**Figure 5:**
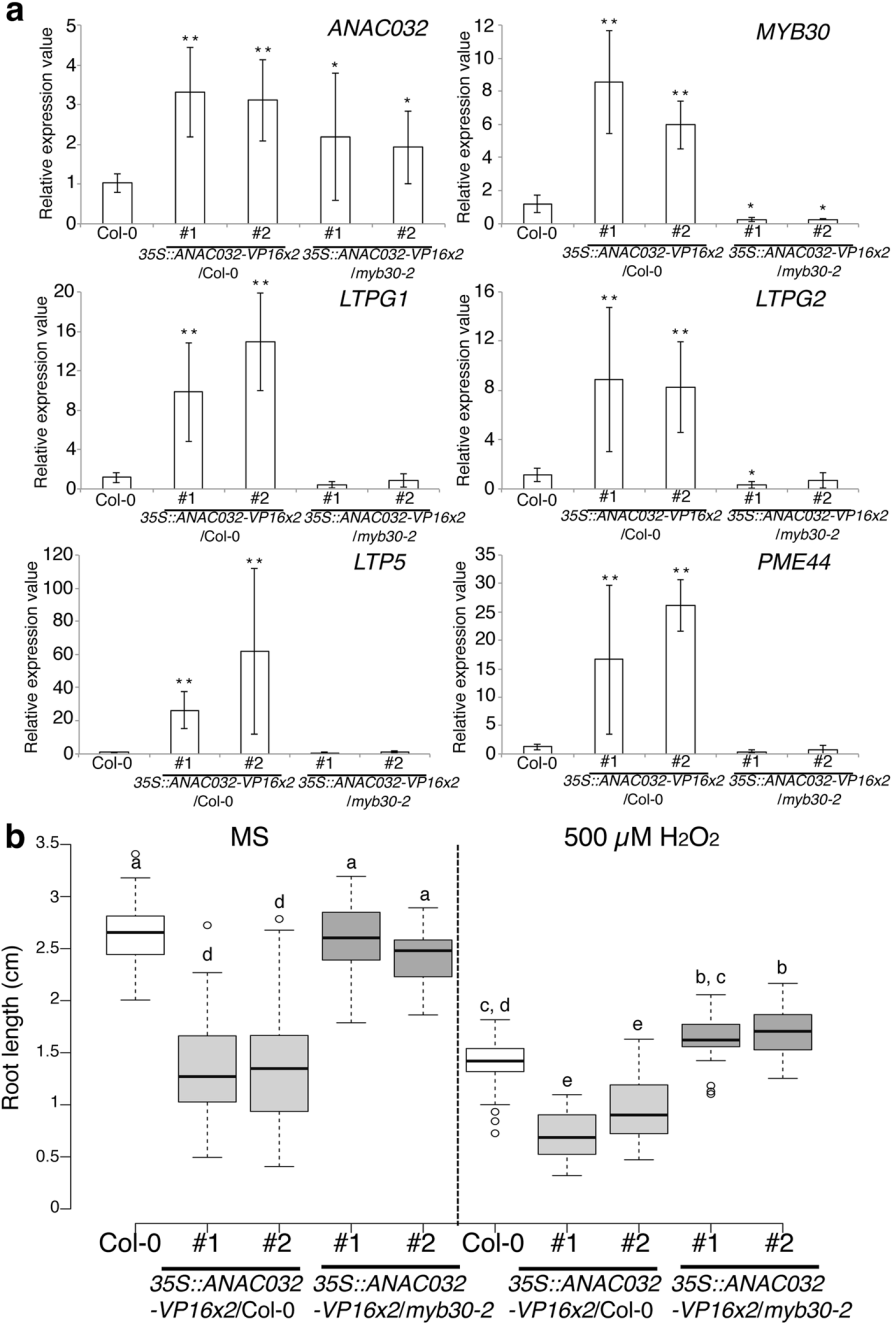
Expression analysis of *ANAC032* and MYB30 target genes in *ANAC032-VP16x2* OX lines and their root length. (**a**) RT-qPCR analysis of *ANAC032* and MYB30 target genes in the 6-day-old whole root of Col-0, *ANAC032-VP16x2* OX in Col-0 line #1 and #2, and *ANAC032-VP16x2* OX in *myb30-2* line #1 and #2 (n = 3: means ± SD). **p < 0.01 and *p < 0.05, determined using Student’s *t*-test compared to expression in Col-0. (**b**) Root length of 5-day-old plants of Col-0 (white box), two independent *35S::ANAC032-VP16x2*/Col-0 lines (grey boxes), and two independent *35S::ANAC032-VP16x2*/*myb30-2* lines (dark grey boxes) treated with Murashige and Skoog (MS) agarose medium and 500 µM H_2_O_2_ containing agarose medium for 24 h (n = 30, letters above boxes indicated statistically significant differences between samples as determined by Tukey’s HSD test (*p* < 0.05)).

The RNAseq analysis indicated that ANAC032 was a candidate for the upstream transcription regulator of *MYB30*. We confirmed this possibility using RT-qPCR (Fig. 5). *MYB30* and its target genes were highly upregulated in *ANAC032-VP16x2* OX lines. To provide further evidence that *MYB30* was regulated by ANAC032, we overexpressed *ANAC032-VP16x2* in *myb30-2* mutants. Compared to *ANAC032-VP16x2* OX in the wild type, *MYB30* and its target genes had significantly lower expression levels in *ANAC032-VP16x2* OX in *myb30-2* mutants (Fig. 5a). This result strongly indicated that the MYB30 regulatory network is regulated by ANAC032. To reveal the importance of the MYB30 regulatory network for root development alongside the ANAC032 gene regulatory network, we measured the root length of *ANAC032-VP16x2* OX in *myb30-2* mutants (Fig. 5b). The root growth inhibition in *ANAC032-VP16x2* OX in *myb30-2* mutants was alleviated compared with Col-0 background under both control and 500 µM H_2_O_2_ treatment. This indicates that ANAC032 inhibits root elongation through the MYB30 gene regulatory network.

**Figure 6:**
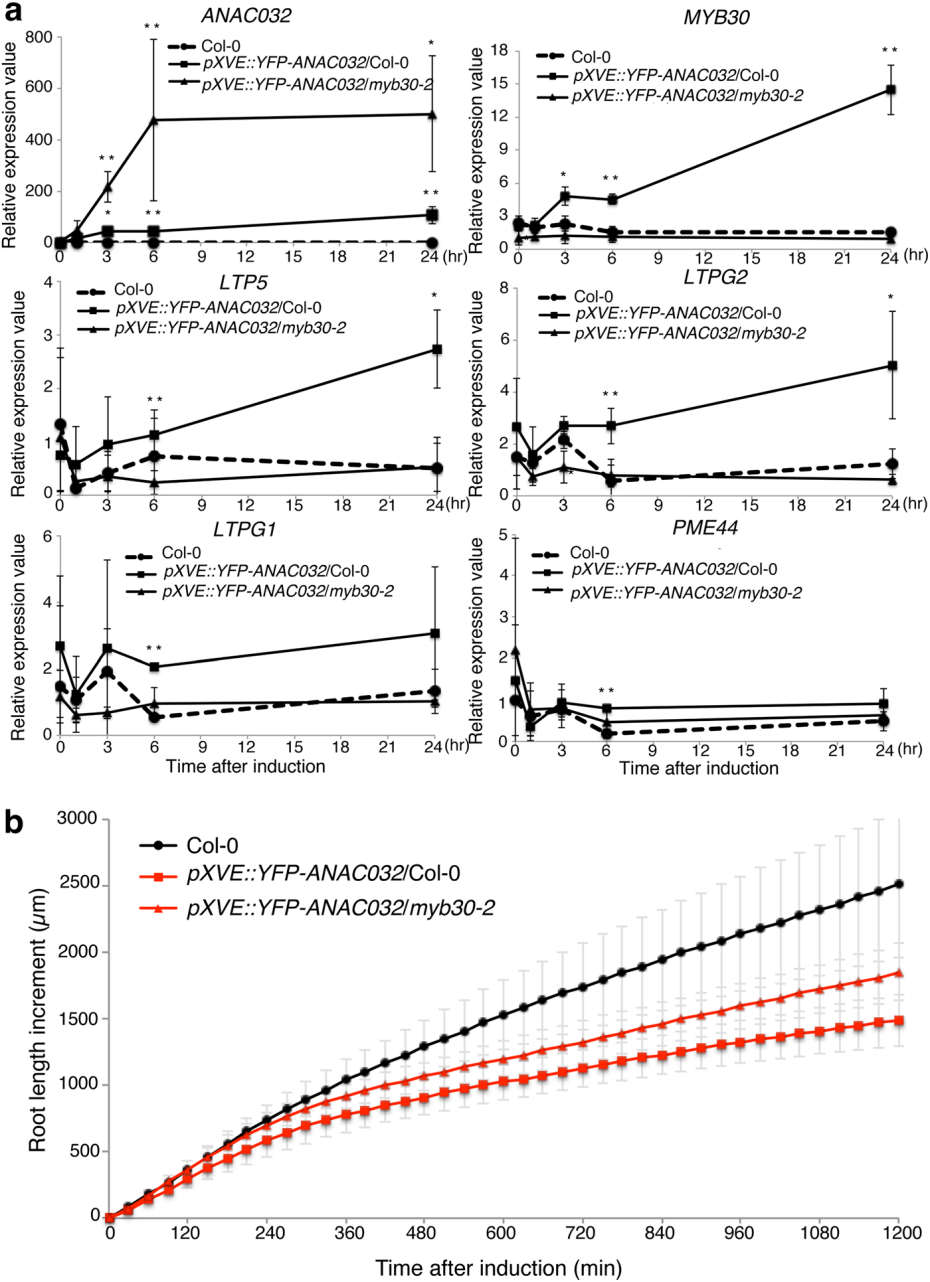
Expression analysis of *ANAC032* and MYB30 target genes in *ANAC032* estradiol-inducible lines and root length increase in estradiol induction. (**a**) Time-course RT-qPCR of *ANAC032* and MYB30 target genes after 0, 1, 3, 6, and 24 h of 5 µM estradiol treatment in the 5-day-old whole root of Col-0 (circle with dashed lines), *pXVE::YFP-ANAC032* in Col-0 (squares with solid lines), and *pXVE::YFP-ANAC032* in *myb30-2* (triangle with solid lines) (n = 3: means ± SD). **p < 0.01 and *p < 0.05, determined using Student’s *t*-test compared with Col-0 at each time point. (**b**) The increase in the length in 5-day-old roots of Col-0 (black circles), *pXVE::YFP-ANAC032* in Col-0 (red squares), and *pXVE::YFP-ANAC032* in *myb30-2* (red triangles) plants treated with 5 µM estradiol containing agarose medium. Root length increase was measured every 30 min after starting time-lapse imaging (n = 10, means ± SD).

We also performed a time course gene expression analysis using *ANAC032* estradiol-inducible line (Fig. 6a). *ANAC032* was strongly induced after 1 h treatment with estradiol. However, *MYB30* was not induced until after 3 h of treatment with estradiol. This time gap between the induction of *ANAC032* and *MYB30* was suggestive of a gene regulatory cascade. Moreover, the MYB30 target genes, *LTPG1*, *LTPG2*, *LTP5*, and *PME44*, were induced after 6 h of treatment with estradiol. We also investigated gene expressions of *MYB30*, *LTP5*, *LTPG1*, *LTPG2*, and *PME44* in the *ANAC032* induction line in *myb30-2* mutants (Fig. 6a). In *myb30-2* mutants, these genes had low expressions throughout the estradiol treatment, even though *ANAC032* was strongly induced. These results also strongly suggested that ANAC032 drove the *MYB30* regulatory gene network. Since *ANAC032* did not fuse with VP16x2 in the inducible line, this result also indicated that ANAC032 was a transcriptional activator. We also confirmed the involvement of the MYB30 regulatory network in the control of root elongation under ANAC032 by using time-lapse imaging (Fig. 6b and Supplementary Movie S3). Root elongation in the *ANAC032* estradiol-inducible line in *myb30-2* mutants exhibited slower elongation and later than that in Col-0 but faster than *ANAC032* induction in Col-0. These results indicated that MYB30 partly contributed to the regulatory network of ANAC032 for root growth regulation.

Our RNAseq results and previous study indicate that ANAC032 is a repressor of the expression of genes associated with flavonoid biosynthesis. We also investigated the expression of flavonoid biosynthesis genes in *ANAC032-VP16x2* OX, in the wild type, and in *myb30-2* (Supplementary Fig. S4). We chose three genes, *PAL*, *CHS*, and *TTG1*, which showed relatively high levels of expression in our RNAseq dataset. The expression of these genes was downregulated in *ANAC032-VP16x2* OX in Col-0. However, the expression of *CHS* and *PAL* were upregulated slightly in *ANAC032-VP16x2* OX in *myb30-2* mutants. These results indicated that flavonoid biosynthesis genes were not directly targeted by ANAC032 and that the MYB30 regulatory network would have a feedback effect on the expression of genes associated with flavonoid biosynthesis in roots.

We also measured the ROS levels in the roots of Col-0, *myb30-2*, *ANAC032-VP16x2* OX in Col-0, *ANAC032-VP16x2* OX in *myb30-2*, *pXVE::YFP-ANAC032* in Col-0, and *pXVE:YFP-ANAC032* in *myb30-2*. ROS levels among Col-0, *myb30-2*, *ANAC032-VP16x2* OX in Col-0, and *ANAC032-VP16x2* OX in *myb30-2* were comparable. Moreover, ROS levels of Col-0, *myb30-2*, *pXVE::YFP-ANAC032* in Col-0, and *pXVE::YFP-ANAC032* in *myb30-2* under both the control and the 5 µM estradiol treatment were also comparable (Supplementary Fig. S5). These results indicated that changes in gene expression in the *ANAC032-VP16x2* OX and *ANAC032* estradiol-inducible lines were not due to altered endogenous ROS levels in the roots.

## Discussion

In the present study, we showed that one important transcription factor, ANAC032, regulates root growth under ROS signalling. *ANAC032* encodes for a NAC-type transcription factor and is a transcriptional activator. Under H_2_O_2_ treatment, *ANAC032* is upregulated and controls downstream gene expression and represses root cell elongation. ROS has been suggested as a candidate growth regulator and signalling molecule. Many studies show that ROS acts as a regulator under both biotic- and abiotic stresses. However, the molecular mechanism underlying ROS signalling and growth regulation remains unclear. To further our understanding of this, our ROS-map is informative for revealing new players in the ROS signalling pathways. We have previously reported that MYB30 is an important transcription factor that links ROS signalling and root development^10^. MYB30 also regulates the expression of genes involved in transporting very long chain fatty acids (VLCFA). The expression of these genes was also shown to be induced in our ROS-map dataset. We chose *ANAC032* because *ANAC032* expression is induced within 1 h of treatment with H_2_O_2_. Moreover, NAC-type transcription factors are known to regulate plant development. There are no apical meristem/NAM and CUP-shaped cotyledon2/CUC2 that are important for controlling development of shoot apical meristems^11–13^. Vascular-related NAC domain 7 (VND7) is known to regulate the maturation of vascular tissue in roots^22^. These studies indicated that the NAC domain containing transcription factors regulates plant development. However, NAC genes form a large gene family with 105 NAC proteins identified in *Arabidopsis*^20^; therefore, it would be difficult to study for a single mutant analysis. Indeed, the root growth of a T-DNA insertion mutant of *ANAC032* (SALK_012253) showed the same response to the H_2_O_2_ treatment as that of the wild type. We decided to analyse ANAC032 function using the constitutive overexpression of ANAC032 fused with the VP16 transcriptional activation domain.

*ANAC032* transcriptional fusion was strongly expressed in the vasculature. Since this expression pattern was very similar to that of *VND7*^22^, we initially considered that ANAC032 would be part of the regulatory circuit of VND7. However, the ANAC032 translational fusion line is strongly nuclear-localised in the epidermis of the elongation zone but is weakly expressed in the vascular tissue compared with that of transcriptional fusion. This might indicate that the ANAC032 protein is regulated after translation or that *ANAC032* mRNA is degraded in the vascular tissue. According to our time-lapse imaging, *ANAC032* is expressed in the transition zone accompanying root development. Moreover, ANAC032 translational fusion started to express from the basal meristematic zone in the primary root after H_2_O_2_ treatment. The transition from cell proliferation to elongation started gradually in the basal meristematic zone^1^. In addition, transcriptome analysis with *ANAC032-VP16x2* OX lines showed that the gene ontology category of cell cycle regulated genes was not significantly enriched. This also indicated that ANAC032 functions in the regulation of cell elongation at the boundary between the basal meristematic and elongation zone. In addition, since *ANAC032* was also expressed in vascular tissue, ANAC032 might have other functions involving the regulation of vascular tissue development like VND7. However, we did not observe the formation of ectopic xylem vessel elements in *ANAC032-VP16x2* OX lines, like those observed during *VND7* overexpression^23^.

Supporting our hypothesis, *ANAC032-VP16x2* OX lines had shorter root lengths and elongation zones than those of the wild type. In addition, the *ANAC032* estradiol-inducible line inhibited root elongation after 360 min of estradiol treatment. In this case, ANAC032 was not fused with the VP16x2 transcriptional activation domain. These results strongly indicate that ANAC032 itself inhibits cell elongation. However, previous research has shown that ANAC032 function switches to that of a transcriptional negative regulator when fused with short transcriptional repressor domain (SRDX) and exhibits the short root phenotype whereas overexpression of *ANAC032* led to normal root growth^17^. Though we should have analysed the *ANAC032-SRDX* lines in our experimental condition, we also considered that the estradiol-inducible lines might be reliable because it is possible to track the rapid effects of the overexpression of *ANAC032* controlled by the estradiol treatment. Expression induction systems would be important for analysing transcription factors because rapid regulation caused by inducible gene overexpression provides a more focused resource for identifying the primary role of a target transcription factor^24^. However, *ANAC032* is overexpressed in all cell types in the estradiol-inducible line. This might cause artificial effects because the original *ANAC032* expressed limited cell types, such as epidermal, in the basal meristematic zone and the elongation zone. To reveal the function of ANAC032 for root growth by genetic analysis, we also need to analyse the *ANAC032* gene disruptant line. We have already obtained the T-DNA insertion mutant for *ANAC032* (SALK_012253). Previous studies have shown that this T-DNA insertion mutant did not exhibit any visible phenotypes^25,26^; thus, we did not detect the root growth phenotype of this mutant under the H_2_O_2_ treatment. To elucidate the effect of loss of function of *ANAC032* in the root, ANAC032-SRDX driven by the *ANAC032* promoter should be investigated in future studies.

By using RNAseq with the *ANAC032-VP16x2* overexpression line, we found that 999 genes were significantly upregulated. Since the expression of many of the genes was affected by *ANAC032-VP16x2* overexpression and endogenous ROS levels were not altered in roots overexpressing *ANAC032*, we considered that ANAC032 had an epistatic interaction with the ROS transcriptional cascade. Among the upregulated genes, we found GO terms representing responses to plant hormones, such as gibberellin, ABA, brassinosteroid, and GO terms representing metabolic enzymes related to WAX synthesis and sugar transfer. Within these genes, we found that *ATAF1* regulates ABA signal transduction and synthesis^27^, *arginine decarboxylase 2*, a limiting enzyme for polyamine synthesis^28^, and *ANAC102*, which is induced by low-oxygen conditions during seed germination^29^.

These results also indicated that ANAC032 regulates responses to plant hormones under abiotic stress conditions. In addition, we found that *MYB30* and its target genes were highly upregulated in our RNAseq dataset. In addition to the expression analysis, the GFP expression patterns of the translational fusion of ANAC032 and translational fusion of MYB30 at the root tip was also quite similar^10^. MYB30 has important functions as a transcription regulator under biotic and abiotic stress condition^10,30,31^. Interestingly, *MYB30* was rapidly induced in the *ANAC032* estradiol-inducible line and its target genes were induced later. This result indicates that *MYB30* is directly targeted by ANAC032. Moreover, about 25% of the upregulated genes in *MYB30* OX were upregulated by *ANAC032-VP16x2* OX. Among them, the GO categories, “lipid transport” and “wax biosynthesis”, were highly enriched. 3-ketoacyl-CoA synthase 6 (CER6^32^), which is a key enzyme for VLCFA synthesis, and six lipid transfer proteins (LTPs) were included. This is consistent with our previous finding that VLCFAs are important regulators of root cell elongation^10^. This also indicated that the MYB30 transcriptional network regulates root growth under stressful conditions that induce ROS as a signalling molecule, and that this process is under the ANAC032 transcriptional cascade. This is consistent with the root growth phenotype of the *ANAC032* estradiol-inducible line. Although the binding of ANAC032 to the *MYB30* promoter region requires further study, there was little expression of the MYB30 target genes in *ANAC032-VP16x2* OX in *myb30-2* and *ANAC032* estradiol-inducible lines in *myb30-2* mutants, further indicating that MYB30 is a target of ANAC032.

It has also been reported that ANAC032 regulates flavonoid synthesis, especially anthocyanin^19^. Our RNAseq results suggested that the expression of genes associated with flavonoid biosynthesis were significantly downregulated. The inhibition of flavonoid synthesis by ANAC032 is interesting because ANAC032 is thought to be a regulator of stress response^17^, and anthocyanins play an important role in stress protection in plants^33^. ANAC032 may have important functions in regulating the balance between plant growth and stress response. However, the gain-of function type analysis cannot exclude the possibility that the overexpression of a transcription factor would affect other physiological processes indirectly. Future studies should elucidate the details of transcriptional regulation by ANAC032 by using time course transcriptome analysis of estradiol *ANAC032* inducible lines and genome wide ANAC032 DNA-binding analysis such as ChIP-seq.

## Materials and Methods

### Plant materials and growth conditions

*Arabidopsis thaliana* Columbia-0 (Col-0) was used as the wild type. The T-DNA insertion line, *anac032* (SALK_012253) was obtained from the SALK collection and the ABRC. All seeds were sterilised by treating them with 1% bleach and 0.05% Triton X-100 for 5 min, and then washing 3 times with sterilised water. Seeds were germinated on MS (Murashige and Skoog, FUJIFILM Wako Pure Chemical, Osaka, Japan) medium supplemented with 1% sucrose and 1% agarose after two days at 4 °C. Plants were grown vertically in a growth chamber (Panasonic, Osaka, Japan) at 22 °C with a 16 h light/8 h dark cycle. For H_2_O_2_ and estradiol treatments, 5-day-old seedlings were transferred onto MS agarose plates containing 500 µM H_2_O_2_ and 5 µM estradiol (FUJIFILM Wako Pure Chemical).

SALK_012253 was genotyped using left-border primers on the T-DNA (LB) and right-side (RP) and left-side (LP) primers on the genome. The list of primers that were used in this study is provided in Supplementary Table S2.

### Plasmid construction and plant transformation

Plasmids were constructed using Gateway cloning technology (Thermo Fisher Scientific, Waltham, MA). Genomic DNA from Col-0 was used as the template for amplification of the 3045 bp upstream regions of *ANAC032* for transcriptional fusion. 5′ dA overhangs were added to the PCR amplicon using Taq polymerase (Takara Bio Inc., Shiga, Japan). It was then cloned into pENTR5′-TOPO (Thermo Fisher Scientific). For *ANAC032* cDNA cloning, *ANAC032* cDNA region was amplified by the forward primer that contained CACC sequences for TOPO cloning and the reverse primer. Then *CACC-cANAC032* fragments were cloned into pENTR/D-TOPO (Thermo Fisher Scientific). For *YFP-ANAC032* cloning, the *ANAC032* cDNA region was amplified using the forward primer and reverse primer that contained the BamHI site just before the *ANAC032* termination codon. Then *ANAC032-BamHI* fragments were cloned into Aor51H1 and the BamHI site of YFP-Aor51H1-BamHI-pDonr201 plasmids^10^. For the *pXVE::YFP-ANAC032* construct, *YFP-ANAC032* containing pDONR201 was cloned into pMDC7^21^ using LR Clonase II (Thermo Fisher Scientific). For the *pANAC032::GFP*, *ANAC032* promoter region containing pENTR5′-TOPO was cloned into R4L1pGWB550^34^ using LR Clonase II.

For *pANAC032::ANAC032-GFP* construct, the *ANAC032* promoter region containing pENTR5′-TOPO and *cANAC032* containing pENTR/D-TOPO were cloned into R4pGWB550^35^ using LR Clonase II. For the *p35S::ANAC032-VP16x2* construct, *VP16x2* DNA fragments were amplified by PCR using the pBS-PRR5-VP vector that contained the *VP16x2* sequence^36^ as a template. Then the *VP16x2* DNA fragment was cloned into the *Aor51*HI site located after the *att*R1-*Cm*^*r*^-*ccd*B-*att*R2 of pUGW2Ω^37^ vector using the In-Fusion^®^ HD Cloning Kit (Takara Bio Inc.). 2×35S-Ω-*att*R1-*Cm*^*r*^-*ccd*B-*att*R2-VP16x2 DNA fragments were amplified by PCR using the pUGW2Ω-VP16x2 vector as a template, and this DNA fragment was cloned into the *Hin*dIII-*Sac*I site of pGWB501^37^ using the In-Fusion^®^ HD cloning Kit. Then *cANAC032* containing pENTR/D-TOPO was cloned into pGWB501 containing *VP16x2* using LR Clonase II. The resulting plasmids (*pANAC032::GFP*, *pANAC032::ANAC032-GFP*, *35S::ANAC032-VP16x2*, and *pXVE::YFP-ANAC032*) were transferred into *Agrobacterium* and used to transform into Col-0, and *myb30-2*. The list of primers that were used in this study is provided in Supplementary Table S2.

### Quantitative real-time RT-PCR

RNA was isolated from whole roots of 5-day-old plants treated with 500 µM H_2_O_2_ (plus a control) for 1 day using the RNeasy plant kit (QIAGEN, Hilden, Germany). For RNA isolation from the root tip, root tips that contained meristematic- and elongation zones were micro-dissected 6-day-old plants^8^. RNeasy micro kit (QIAGEN) was then used for the RNA isolation. First strand cDNA was synthesised with ReverTra Ace qPCR RT Master Mix with gDNA Remover (TOYOBO, Osaka, Japan). Quantitative real-time PCR (RT-qPCR) was performed using the THUNDERBIRD SYBR qPCR Mix (TOYOBO) on an illumine Eco (Illumina, San Diego, CA). The list of primers that were used in this study is provided in Supplementary Table S2. Primers information of *MYB30*, *LTPG1*, *LTPG2*, *LTP5*, and *PME44* are described in Mabuchi *et al*.^10^. Primers information of *CHS*, *PAL*, and *TTG1* are described in Mahmood *et al*.^18^. RT-qPCR efficiency and the CT value were determined using the standard curves for each primer set. Efficiency corrected transcript values of three biological replicates for all samples were used for determining the relative expression values. The level of each value was normalised against the level of *PDF2*^38^.

### Semi-quantitative RT-PCR

Semi-quantitative RT-PCR was performed using KOD-One DNA polymerase (TOYOBO) with the forward and reverse primer set that was designed to amplify the whole *ANAC032* coding sequence. *PDF2* was used as the internal control gene. A list of the primers used in this study is provided in Supplementary Table S2.

### RNAseq experiments

The cDNA libraries were generated from 100 ng of total RNA samples using a TrueSeq RNA sample preparation kit (Illumina) as described previously^39^, and the amount of cDNA was determined by PhiX Control (Illumina). Both ends of the cDNA libraries were sequenced for 60 cycles using a paired-end module. Two biological replicates were conducted for each experiment.

### RNAseq data analysis

The short read results from sequencing were mapped onto the *Arabidopsis* genome (TAIR10: http://www.arabidopsis.org/) in the Bowtie software^40^. Then these datasets were normalised and a false discovery rate (FDR) and a fold change (FC) were calculated using the edgeR package for R^41^. We used the following cut off values to determine differentially expressed genes among Col-0 and *35S::ANAC032-VP16x2/Col-0* T_2_; |FC| > 2 and FDR q < 0.0001. The data were deposited in DDBJ Sequence Read Archive (DRA) at the DNA Data Bank (DDBJ; http://www.ddbj.nig.ac.jp) with accession number DRA008125. Enriched GO categories were analysed by ChipEnrich software^42^. The TMeV program (http://www.tigr.org) was used for visualising of the heat map for enriched GO categories and fold changes of expression of transcription.

### Phenotypic and microscopic analyses

For measuring the whole root length, roots were scanned using a flatbed scanner GT-7400U (EPSON, Nagano, Japan) while growing on plates. Root length was measured by importing images into Image-J. Laser scanning confocal microscopy for *pANAC032::GFP*/Col-0 and *pANAC032::cANAC032-GFP*/Col-0 was performed with a ZEISS LSM700 system (ZEISS, Oberkochen, Germany) on propidium iodide (PI) stained roots at objectives of 20×. Roots were stained with PI in a 10 µg/ml dilution in water for 3–5 min with 488 nm excitation and 500–550 nm emission for GFP, 555 nm excitation with 580–680 nm emission for PI. Images of roots were taken and connected using the tile scan mode in the ZEN software (ZEISS). 10 Z-stack images of *pANAC032::cANAC032-GFP*/Col-0 were taken with a Leica SP8 system (Leica Camera AG, Wetzlar, Germany) of PI stained roots. With 488 nm excitation and 490–543 nm emission for GFP, 552 nm excitation with 583–742 nm emission for PI. The maximum projection was created by the LAS X software (Leica Camera AG). For time-lapse imaging, a Lab-Tek Chambered Coverglass w/cvr (Thermo Fisher Scientific) was used as described previously^10^. The chamber containing the plants were imaged with a confocal microscopy Leica SP8 system (for *pANAC032::cANAC032-GFP*/Col-0) and a fluorescence microscope DMI 6000B-AFC (Leica Camera AG) (for *pXVE::YFP-cANAC032*) at objectives of 20x. Time-lapse images were taken by the LAS X every 30 min for 20 h. Assembling of images were also done with LAS X. The root elongation rate was measured by image-J software on time series consisting of images that had been taken every 30 min.

Quantifying the GFP intensity of confocal images was based on previously described methods^43^. The confocal images were analysed with Image-J by drawing a rectangle of 200 µm wide and 25 µm height starting from the end of the last columella and extending until the end of the elongation zone. The means of the signal were quantified with the “Plot Profile” option. For the pseudo-colour integrative surface plot, time-lapse images were analysed with the Image-J plugin “interactive 3D surface plot”.

### ROS measurements

ROS production was measured using L-012-mediated chemiluminescence as described previously^10^. For the estradiol treatment, 5 µM estradiol (final concentration) was added to a 100 µM L-012-containing reaction mix (FUJIFILM Wako Pure Chemical). Luminescence was measured every 3 min for 20 h using a microplate reader (Spark 10 M; Tecan, Männedorf, Switzerland).

## Supplementary information

Supplementary information

Supplementary Movie S1

Supplementary Movie S2

Supplementary Movie S3

Supplementary Table S1

## Data Availability

The sequencing data have been deposited in DDBJ Sequence Read Archive (DRA) at the DNA Data Bank (DDBJ; http://www.ddbj.nig.ac.jp) under accession code: DRA008125.
