# Supplementary material for: ANAC032 regulates root growth through the MYB30 gene regulatory network
Source: Sci Rep. 2019 Aug 6;9:11358. doi: 10.1038/s41598-019-47822-0 (PMC6684591; doi:10.1038/s41598-019-47822-0)
Supplement: Supplementary file 1 — Supplementary information [file 41598_2019_47822_MOESM1_ESM.pdf]

Supplementary information

ANAC032 regulates root growth through the MYB30 gene regulatory network

Hiromasa Maki, Satomi Sakaoka, Tomotaka Itaya, Takamasa Suzuki, Kaho Mabuchi, Takashi Amabe, Nobutaka Suzuki, Tetsuya Higashiyama, Yasuomi Tada, Tsuyoshi Nakagawa, Atsushi Morikami, Hironaka Tsukagoshi*

*Corresponding Author: thiro@meijo-u.ac.jp

This PDF file includes:
Supplementary Figure S1 to S5
Supplementary Table S2

Supplementary Data file includes:
Supplementary Table S1. List of significantly differentially expression genes between Col-0 and *ANAC032-VP16x2* OX.

Supplementary Movie file includes:

Supplementary Movie S1. Time-lapse imaging of *pANAC032::ANAC032-GFP*/Col-0. Left and right movies show 5-day-old *pANAC032::ANAC032-GFP*/Col-0 roots treated with Murashige and Skoog (MS) agarose medium and 500 μM H_2O_2 containing agarose medium, respectively. The images were taken under a confocal microscope every 20 min for 20 h. Scale Bar = 250 μm.

Supplementary Movie S2. Pseudo-colour GFP intensity of the root from supplementary Movie S1. Integrative 3D surface plot image of *pANAC032::ANAC032-GFP*/Col-0 roots treated with MS Murashige and Skoog (MS) agarose medium and 500 μM H_2O_2 containing agarose medium. X axis, Y axis, and Z axis represent the width of the movies, distance from the top of images, and GFP fluorescent intensity, respectively.

Supplementary Movie S3. Time-lapse imaging of ANAC032 estradiol inducible lines. Time lapse imaging of Col-0 (two roots from the bottom), *YFP-ANAC032* inducible lines in Col-0 background (three roots in the middle), and *YFP-ANAC032* in *myb30-2* mutant lines (three roots from the top) treated with 5 μM estradiol containing agarose medium. The images were taken under a fluorescence microscope every 30 min for 20 h.

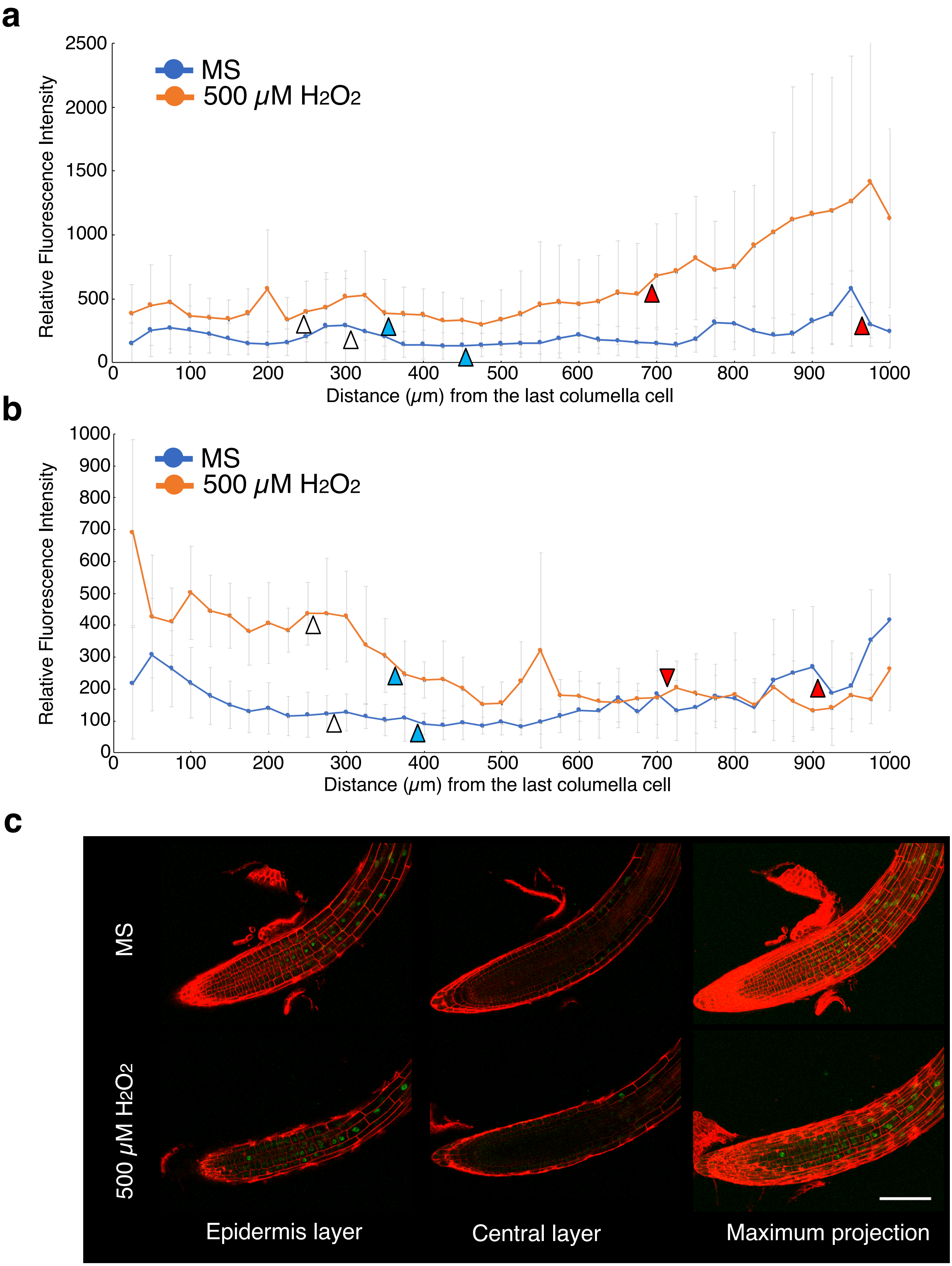
a
MS
500 μM H2O2
Relative Fluorescence Intensity
2500
2000
1500
1000
500
0
0 100 200 300 400 500 600 700 800 900 1000
Distance (μm) from the last columella cell
b
MS
500 μM H2O2
Relative Fluorescence Intensity
1000
900
800
700
600
500
400
300
200
100
0
0 100 200 300 400 500 600 700 800 900 1000
Distance (μm) from the last columella cell
c
MS
500 μM H2O2
Epidermis layer
Central layer
Maximum projection

Supplementary Figure S1. ***pANAC::GFP*** **and** ***pANAC::ANAC032-GFP*** **expression in the root.** (a) Relative GFP fluorescence intensity of 5-day-old *pANAC032::GFP* roots that were treated with Murashige and Skoog (MS) agarose medium (blue line) and 500 µM H_2O_2 containing agarose medium (orange line) for 24 h. White arrow heads, blue arrow heads, and red arrow heads in the line graph indicate the average length from the first columella cell of the end of the apical meristematic zone (MS; 310 µm, H_2O_2: 249 µm), the end of the basal meristematic zone (MS; 453 µm, H_2O_2: 354 µm), and the end of the elongation zone (MS; 971 µm, H_2O_2: 695 µm), respectively (n = 5, means ± SD). (b) Relative GFP fluorescence intensity of 5-day-old *pANAC032::ANAC032-GFP* roots that were treated with MS agarose medium (blue line) and 500 µM H_2O_2 containing agarose medium (orange line) for 24 h. White arrow heads, blue arrow heads, and red arrow heads in the line graph indicate the average length from the first columella cell of the end of the apical meristematic zone (MS; 280 µm, H_2O_2: 259 µm), the end of the basal meristematic zone (MS; 396 µm, H_2O_2: 364 µm), and the end of the elongation zone (MS; 906 µm, H_2O_2: 712 µm), respectively (n = 5, means ± SD). (c) Ten Z-stack images of the 5-day-old *pANAC032::ANAC032-GFP* root tips that were treated with MS agarose medium and 500 µM H_2O_2 containing agarose medium for 24 h. Epidermis layer, central layer, and maximum projection indicate the images from the root epidermis layer, number 5 layer, and maximum projection from all 10 Z-stack images, respectively. Scale bar = 100 µm.

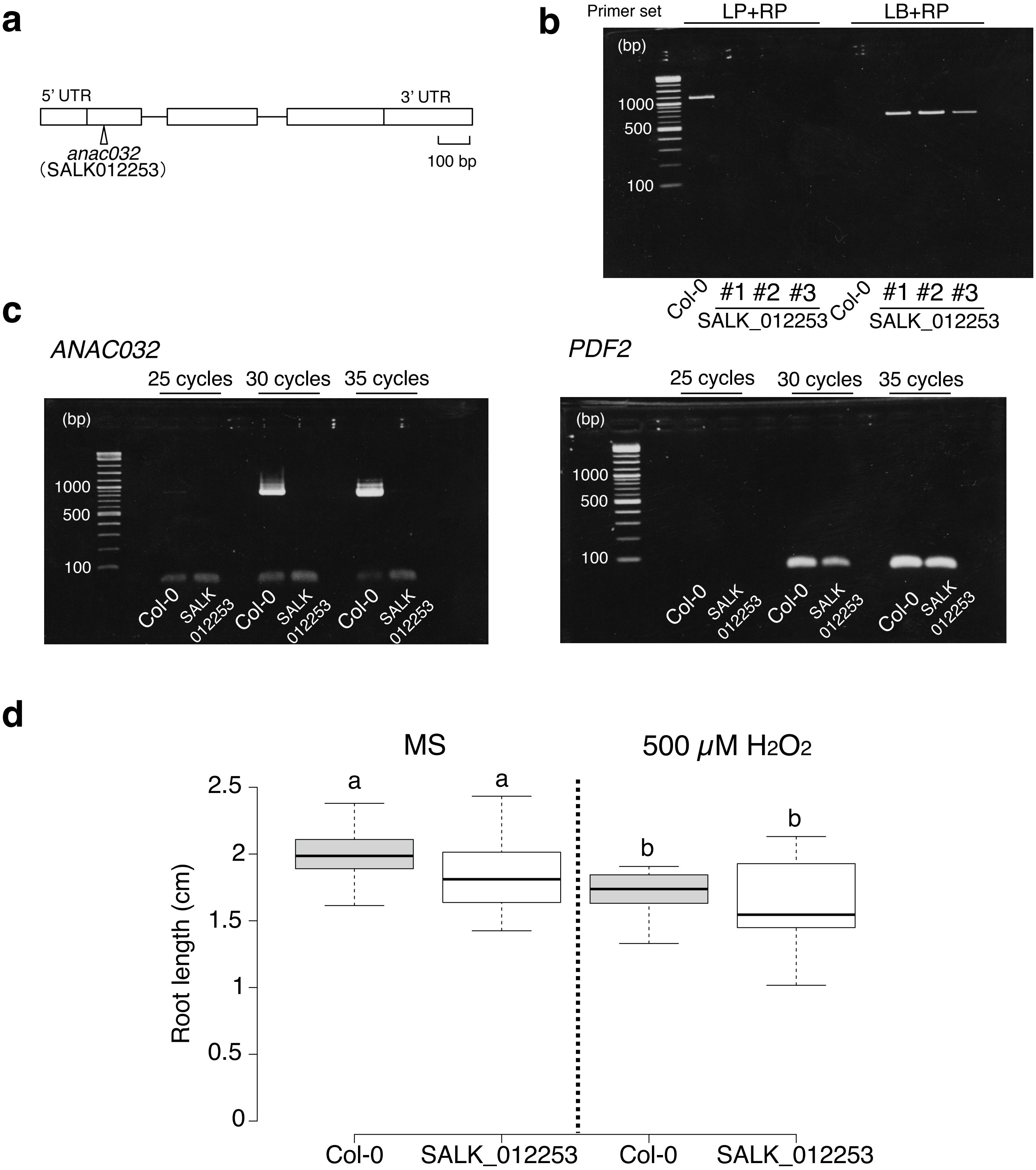

a
5' UTR
3' UTR
anac032
(SALK012253)
100 bp
b
Primer set
LP+RP
LB+RP
(bp)
1000
500
100
Col-0
#1 #2 #3
SALK_012253
Col-0
#1 #2 #3
SALK_012253
c
ANAC032
25 cycles
30 cycles
35 cycles
(bp)
1000
500
100
Col-0
SALK 012253
PDF2
25 cycles
30 cycles
35 cycles
(bp)
1000
500
100
Col-0
SALK 012253
d
MS
500 μM H2O2
Root length (cm)
2.5
2
1.5
1
0.5
0
a
a
b
b
Col-0
SALK_012253
Col-0
SALK_012253

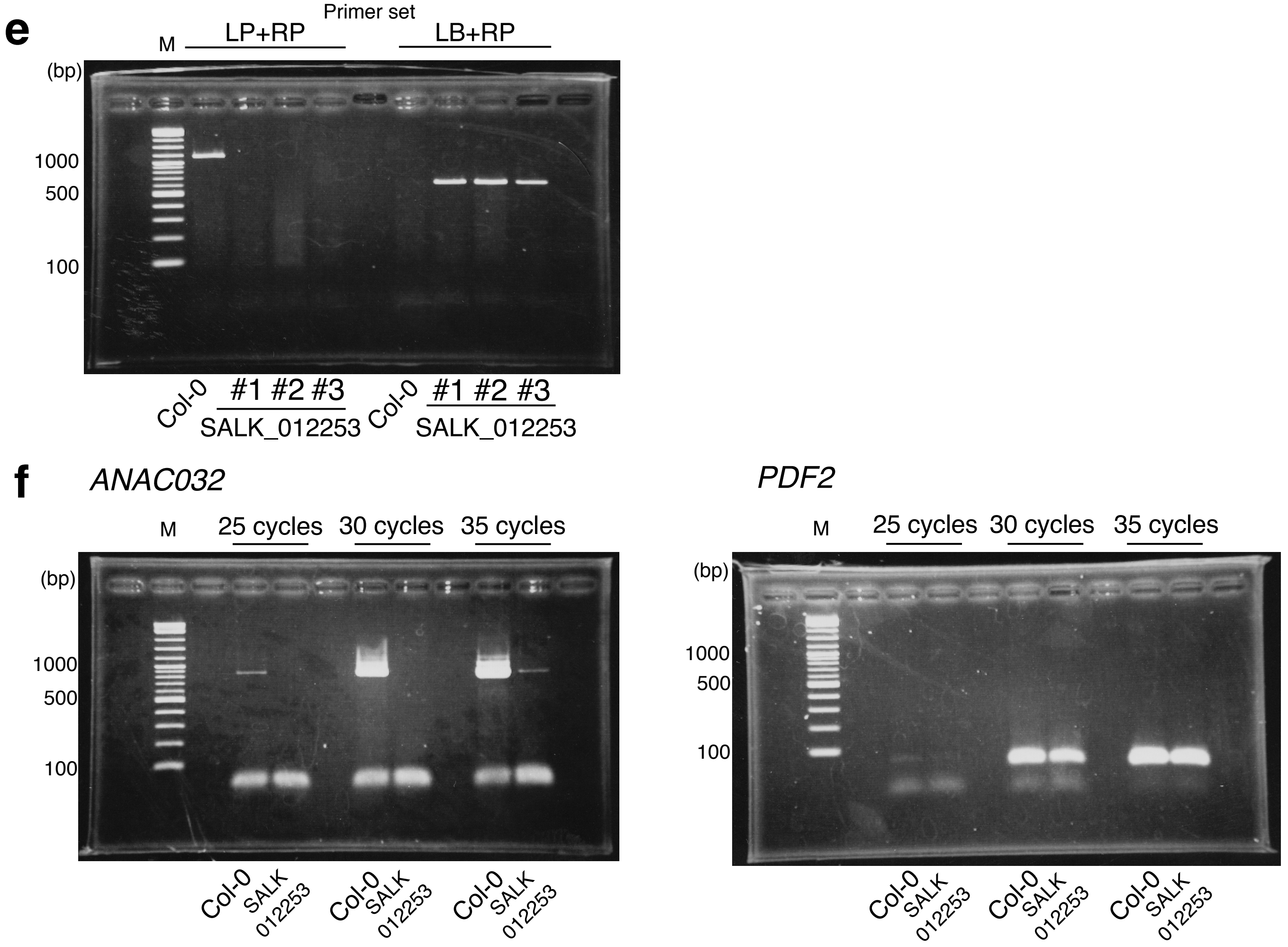

Supplementary Figure S2. ***ANAC032*** **expression level and root phenotypes of the T-DNA insertion line for** ***ANAC032*****.** (a) Genomic structure of *ANAC032* and the position of T-DNA in SALK_012253. (b) Genotyping results of SALK_012253 T-DNA insertion line. We performed genomic PCR with a pair of gene-specific primers (primer set: LP+RP, PCR amplicon size was about 1100 bp) and a combination of a gene-specific primer and a T-DNA left border-specific primer (primer set: LB+RP, PCR amplicon size was about 650 bp). One Col-0 and 3 independent SALK_012253 T-DNA insertion lines (#1, #2, and #3) were analysed. (c) Semiquantitative RT-PCR for detecting full length of *ANAC032* coding sequence from Col-0 and SALK_012253. *PDF2* served as a control. PCR was performed with 25, 30, and 35 cycles. *ANAC032* coding sequence was 762 bp, and amplicon from *PDF2* primer set was 61 bp. (d) Root length of 5-day-old plants of Col-0 (white box) and SALK_012253 (grey boxes) treated with Murashige and Skoog (MS) agarose medium and 500 µM H_2O_2 containing agarose medium for 24 h (n = 30, letters above boxes indicate statistically significant differences between samples as determined by Tukey's HSD test ($p < 0.05$)). (e) Full agarose and longer exposure gel image for the supplementary figure S2 (b). (f) Full agarose and longer exposure gel images for the supplementary figure S2 (c). M, DNA molecular maker.

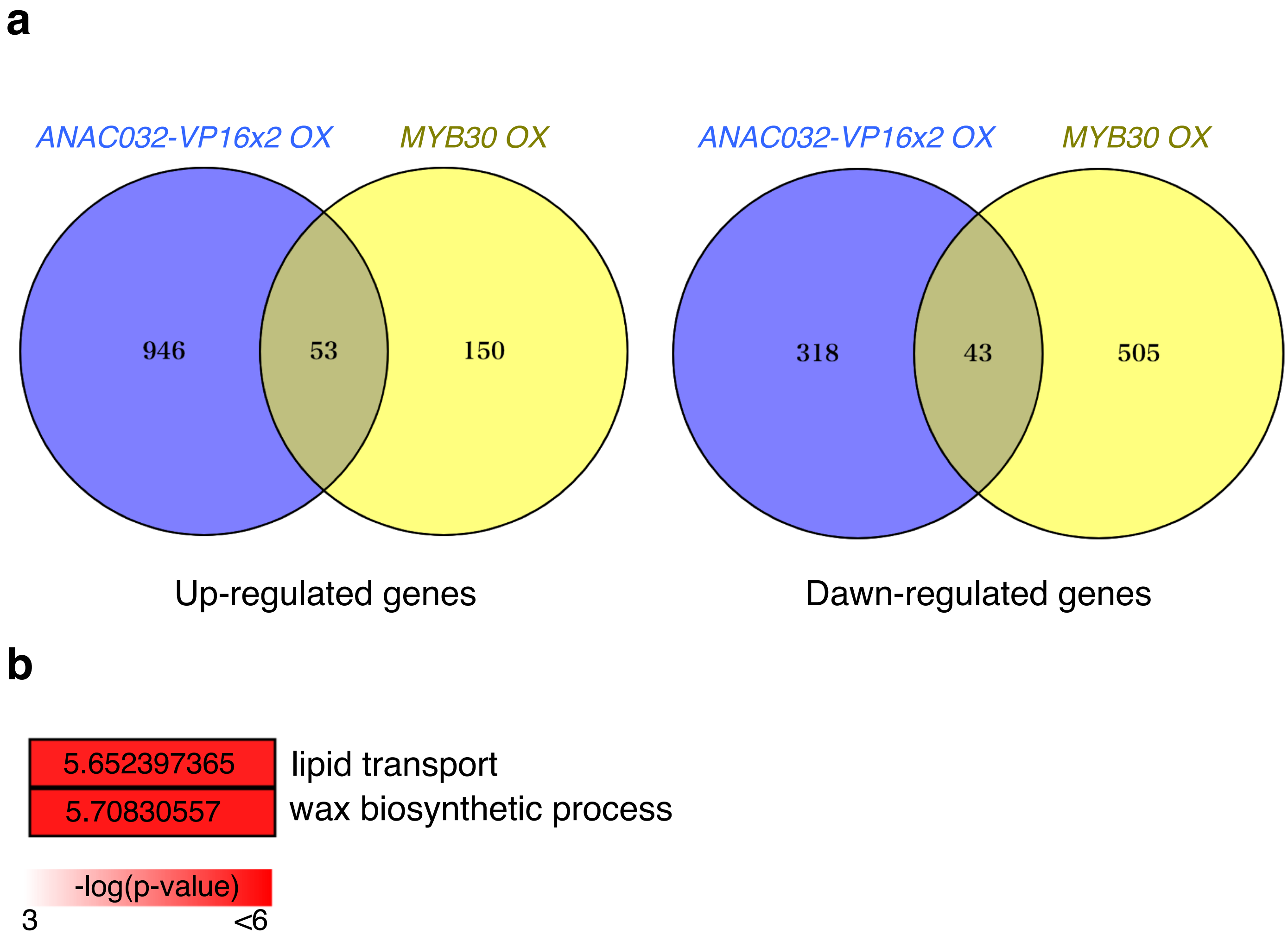

Supplementary Figure S3. Genes co-regulated by *ANAC032-VP16x2* OX and *MYB30* OX. (a) Venn diagrams of the significantly upregulated and downregulated genes in the *ANAC032-VP16x2* OX (blue) and *MYB30* OX (yellow) in the RNAseq datasets. Gene list in *MYB30* OX was retrieved from previous paper (Mabuchi et al., 2018). (b) Gene Ontology categories enriched among genes significantly upregulated both in *ANAC032-VP16x2* OX and *MYB30* OX.

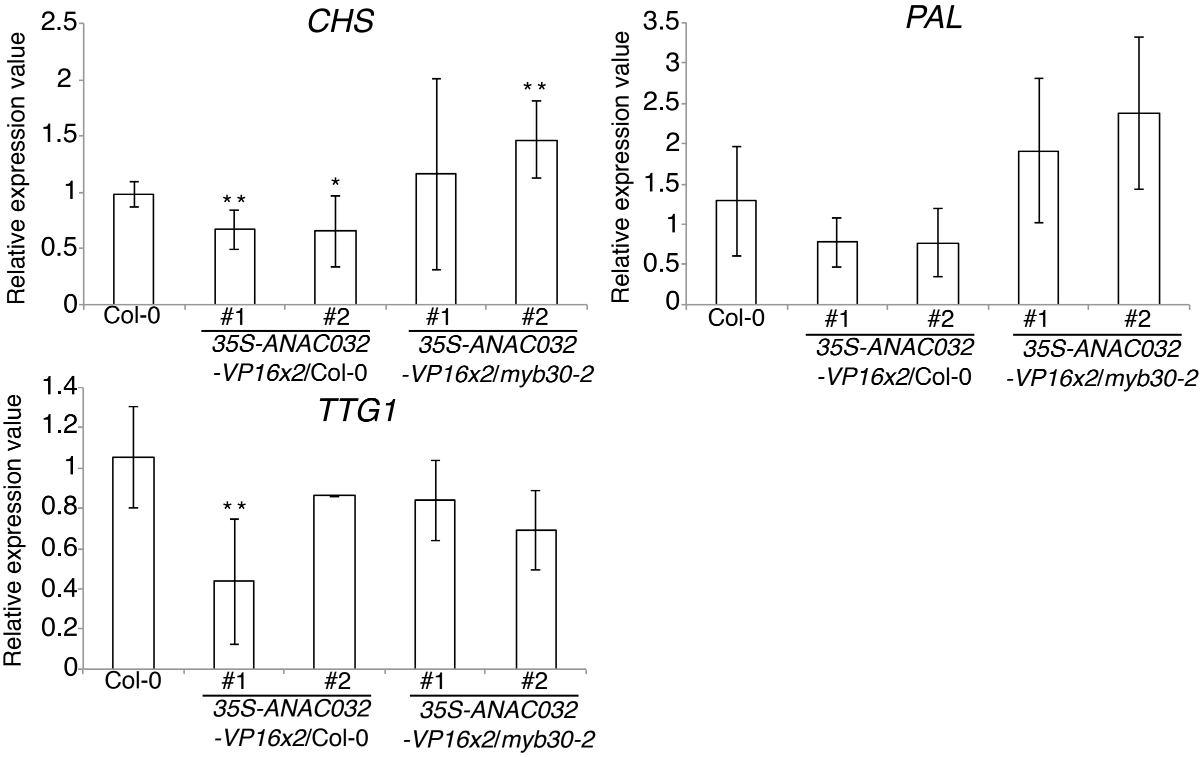

Supplementary Figure S4. Expression analysis of flavonoid biosynthesis genes in *ANAC032-VP16x2* OX lines. RT-qPCR analysis of *CHS*, *PAL*, and *TTG1* in the 6-day-old whole root of Col-0, *ANAC032-VP16x2* OX in Col-0 line #1 and #2, and *ANAC032-VP16x2* OX in *myb30-2* line #1 and #2 (n = 3: means ± SD). $^{**}p < 0.01$ and $^{*}p < 0.05$, determined using Student's *t*-test compared to expression in Col-0.

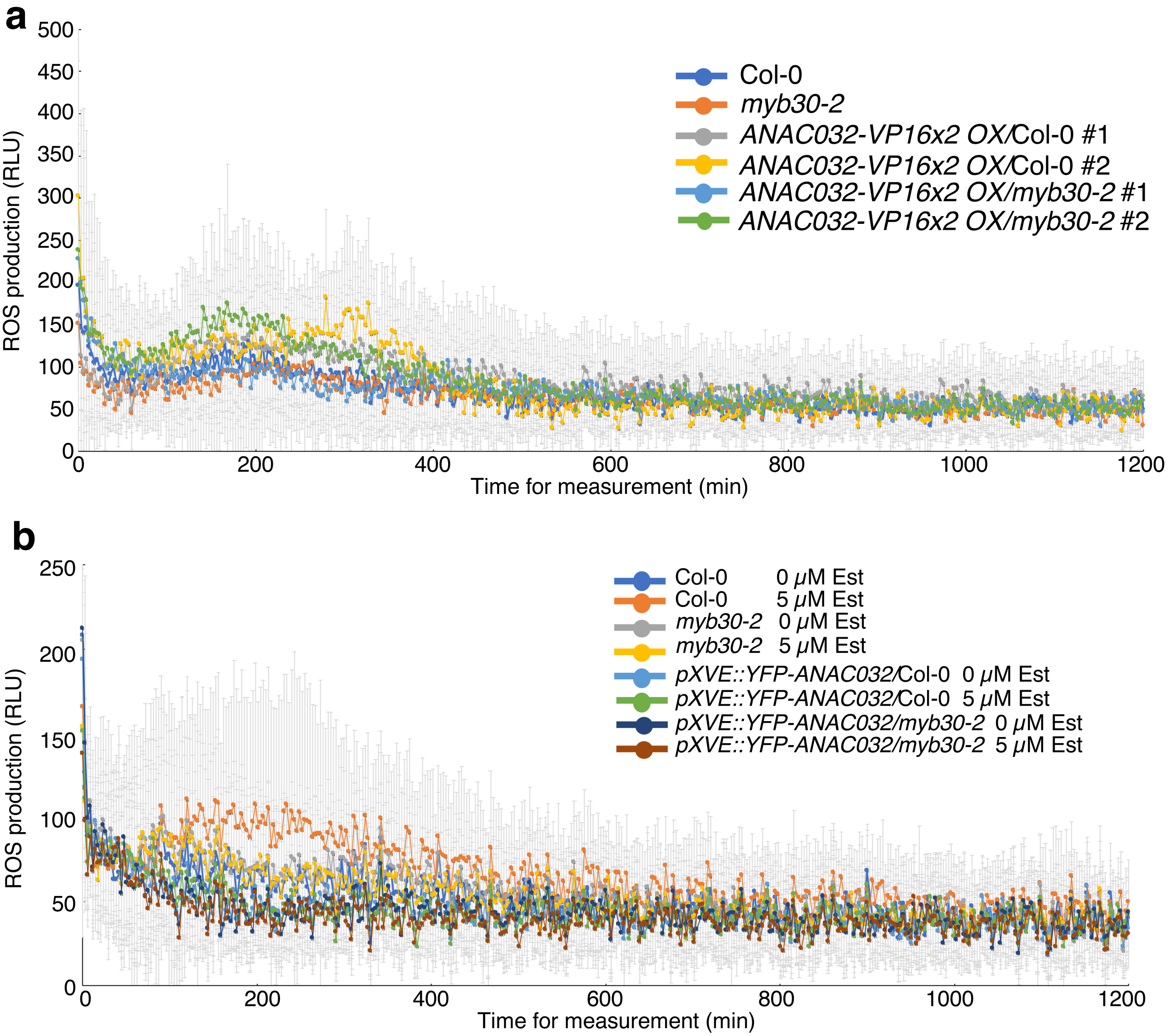

Supplementary Figure S5. Chemiluminescence assay for measuring endogenous ROS production of the variety of roots. (a) ROS measurements of 6-day-old Col-0 roots, *myb30-2* roots, *ANAC032-VP16x2* OX in Col-0 line #1 and #2 roots, and *ANAC032-VP16x2* OX in *myb30-2* line #1 and #2 roots (n = 12: means ± SD). RLU: relative luminescence units. (b) ROS measurements of Col-0 roots, *myb30-2* roots, *pXVE::YFP-ANAC032* in Col-0 roots, and *pXVE::YFP-ANAC032* in *myb30-2* roots mock-treated or treated with 5 μM estradiol (n = 20: means ± SD).

Supplementary Table S2. Primers used in this study.

Name	Sequence (5'-3')
Pro_ANAC032-F	TCTGATATCACATATCTGCTTTTGTC
Pro_ANAC032-R	AATTAACCTATTTTCCCCTTTATTGTTGTT
cANAC032-F	CACCATGATGAAATCTGGGGCTGAT
cANAC032-R	GAAAGTTCCCTGCCTAACCACAAGT
cANAC032_YFP-F	ATGATGAAATCTGGGGCTGAT
cANAC032_BamHI-R	CCGGATCCGGTTAGAAAGTTCCCTGCCTAACC
Aor51HI_VP-F	GTGGTTGATAACAGCATGGCCCCCCCGACCGATGTCAGCCT
Aor51HI_VP-R	ATTCGAGCTCTAAGCCCCACCGTACTCGTCAA
pUG2_pGWB501_Hind-F	TCTAGACCCAAGCTTGCATGCCTGCAGG
pUG2_pGWB501_Sac-R	GGGAAATTCGAGCTCTAAGCGCTGTTAT
ANAC032_qPCR-F	TGACTGACACGTGTCCACCGGAAT
ANAC032_qPCR-R	TTACTCAACCGACCGTTGCTACACG
LP_SALK012253_genotype	ACCAACAATTGTGGAAGCAAG
RP_SALK012253_genotype	CTCTCCATTTGGAGGTTTTCC
LB_SALK_T-DNA_detection	TTTCGCCTGCTGGGGCAAACCAG
ANAC032cds_RT-PCR-F	ATGATGAAATCTGGGGCTGATTTGCA
ANAC032cds_RT-PCR-R	TCAGAAAGTTCCCTGCCTAACCACA
